# Removal of drug delivered via a central venous catheter by a dual-lumen haemodiafiltration catheter inserted at the same site: a quantitative flow model

**DOI:** 10.1186/cc9529

**Published:** 2011-03-11

**Authors:** R Kam, JM Mari, TJ Wigmore

**Affiliations:** 1 Imperial College London, UK; 2 INSERM, Lyon, France; 3 Royal Marsden Hospital, London, UK

## Introduction

The objectives of this study were to model and visualise flow in a central vein during continuous venovenous haemodiafiltration (HDF), to measure drug removal when an HDF catheter is co-located with a central venous catheter (CVC) infusing medication. Dual lumen HDF catheters are commonly used to deliver continuous venovenous renal replacement therapy in critical care. These catheters are often co-located with a CVC used to infuse drugs, with the tips lying in close approximation in a great vein. The effect of this co-location on drug delivery to the patient due to aspiration by the HDF machine may be of serious import, with the elimination of important vasoactive drugs or minimally protein-bound antibiotics just two possibilities. This effect has never been studied.

## Methods

A model of a human central vein was constructed using transparent polyvinyl chloride piping. A CVC and an HDF catheter were inserted into this and water flow in the central vein and extracorporeal circuit was generated by centrifugal pumps at physiological volume flow rates. Ink was used as a visual tracer and creatinine solution as a quantifiable tracer to determine the extent of removal of CVC infusate via the HDF catheter. The longitudinal distance of the CVC infusion point from the arterial port of the HDF catheter was altered to quantify its effect on tracer removal.

## Results

Volume flow rates of 1.45 l/minute and 200 ml/minute were achieved in the central vein model and the HDF circuit model, respectively, with laminar flow in the central vein confirmed by Duplex imaging and ink flow analysis. All visible ink and 100% of creatinine tracer infused via the CVC were aspirated by the HDF catheter unless the point of infusion was ≥1 cm downstream of the proximal aspect of the arterial port. No measurable tracer was aspirated when the infusion was ≥2 cm downstream. Orientation of side ports did not significantly affect tracer removal.

## Conclusions

This initial study suggests that drugs infused via a CVC co-located with an in-use HDF catheter may be completely and immediately aspirated into the extracorporeal circuit. This phenomenon could lead to significant drug underdosing with potentially severely deleterious consequences for patients. When co-location cannot be avoided, drugs with important immediate effects or high membrane clearance should be infused sufficiently distal to the inlet of an adjacent HDF catheter.

